# Heroin Regulates Orbitofrontal Circular RNAs

**DOI:** 10.3390/ijms23031453

**Published:** 2022-01-27

**Authors:** Gabriele Floris, Aria Gillespie, Mary Tresa Zanda, Konrad R. Dabrowski, Stephanie E. Sillivan

**Affiliations:** 1 Center for Substance Abuse Research, Lewis Katz School of Medicine, Temple University, Philadelphia, PA 19140, USA; gabriele.floris@temple.edu (G.F.); aria.gillespie@temple.edu (A.G.); mary.tresa.zanda@temple.edu (M.T.Z.); Konrad.dabrowski@temple.edu (K.R.D.); 2 Department of Neural Sciences, Lewis Katz School of Medicine, Temple University, Philadelphia, PA 19140, USA; 3 Department of Biological Sciences, Temple University, Philadelphia, PA 19122, USA

**Keywords:** opioids, self-administration, noncoding RNA, circular RNA

## Abstract

The number of drug overdose deaths involving opioids continues to rise in the United States. Many patients with opioid use disorder (OUD) that seek treatment still experience relapse. Perseverant opioid seeking behaviors represent a major challenge to treating OUD and additional therapeutic development will require insight into opioid-induced neurobiological adaptations. In this study, we explored the regulation of a novel class of RNAs, circular RNAs (circRNAs), by the addictive opioid heroin in the rat orbitofrontal cortex (OFC), a brain region that mediates behavioral responses to rewarding stimuli. Microarray analysis identified 76 OFC circRNAs significantly regulated in male rats after heroin self-administration. We evaluated the specificity of these findings by measuring heroin-associated circRNA expression in female rats after heroin self-administration and in rats that self-administered sucrose. We identify circGrin2b, circUbe2cp, circAnks1a, circAdcy5 and circSlc24A2 as heroin-responsive circRNAs in the OFC. Linear mRNA levels of heroin-associated circRNAs were unchanged except for *Grin2b* and *Adcy5*. An integrated bioinformatics analysis of regulated circRNAs identified microRNAs predicted to bind heroin-associated circRNAs and downstream targets of circRNA: microRNA sponging. Thus, heroin regulates the expression of OFC RNA splice variants that circularize and may impact cellular processes that contribute to the neurobiological adaptations that arise from chronic heroin exposure.

## 1. Introduction

Opioid overdose and deaths continue to rise in the United States, where nearly 190 people die every day from opioid intoxication [1]. Commonly used current pharmacological therapies to manage opioid dependence include buprenorphine and methadone, which both target the mu-opioid receptor [2,3]. However, chronic opioid use induces neurobiological adaptations which extend far beyond the opioid receptor [4]. Understanding the cellular and molecular pathways dysregulated by opioid exposure will provide insight into non-mu-opioid receptor targets that may be identified for more comprehensive treatment of OUD. To address this crucial issue, this study explored expression patterns of a novel class of RNA, circular RNAs (circRNAs), that are regulated in response to self-administration of the opioid heroin in a rat model of drug seeking. Although circRNAs have likely existed for a vast period of time [5], their presence and function in the nervous system have only recently been described [6,7,8,9,10]. In the context of opioid seeking, their role in drug-induced neuroadaptations is understudied and, for the majority of circRNAs, a complete mystery. Exploration of circRNAs in opioid exposure models provides a unique body of information that may inform substance abuse researchers of entirely unknown molecular signaling cascades associated with the critically complex and important phenotypes of opioid seeking. 

CircRNAs are produced from pre-mRNA via non-canonical back-splicing events, during which the 3′ donor portion of an RNA exon attacks the 5′ region of an acceptor RNA exon [11]. This process is likely mediated by RNA-binding proteins (RBPs), such as Fus, Qki and Adar1, and results in a circular RNA product with covalently bound 3′-5′ ends that lacks both a 5′ cap and a poly-A tail [12,13,14]. The lack of a poly-A tail, as well as RNase resistance, increases the stability and longevity of circRNAs compared to mRNA [5]. While many circRNAs are purely exonal and noncoding [15], variation exists, with other circRNAs containing intronic portions [16]. Some exonic circRNAs even contain regions that are capable of protein-coding [17,18]. circRNAs are believed to have numerous cellular functions, ranging from regulation of linear parent mRNA transcription and protein translation to microRNA (miRNA) sponging and sequestration of RBPs [19]. Expression of circRNAs and linear transcripts from the same gene can differ. Sometimes the expression of circRNAs can exceed levels of their linear mRNA counterparts and the brain’s repertoire of circRNAs dramatically increases from birth to adulthood [15,20]. This suggests that circRNAs have a meaningful presence in the cell that can affect different biological processes in a sequence-specific manner and their expression correlates with an increase in cognitive ability [21]. Their effects may be long-lasting and such complexity may subserve neuropsychiatric conditions that are enduring over time. Simply put, circRNA capability is wide-ranging yet ambiguous. 

Brain-enriched, synaptosomal circRNAs are present in the nervous system and many circRNAs are conserved from humans to rodents [6,21]. Therefore, circRNAs are poised to contribute to synaptic function in the brain and, in turn, behavioral output that results from synaptic transmission [6]. Three translational studies have reported aberrant circRNA expression in human neurological diseases and demonstrated in vivo rodent brain manipulation of circRNAs can rescue depression-like behavior in a model of chronic unpredictable stress as well as cognitive behavior and infarct volume in a cerebral focal ischemia model [7,9,22]. Thus, alteration of circRNA expression is sufficient to alter animal behavior and cognition. In the context of opioid exposure, a conserved circRNA from the mu-opioid receptor (Oprm1) is significantly increased after chronic morphine in mice and morphine alters the circRNA profile in the spinal cord, indicating that opioids induce a change in the circRNA profile [23]. Additionally, RNA-sequencing of circRNAs from the nucleus accumbens following conditioned place preference (CPP) described the contribution of a circTmeff-1-mediated pathway to the incubation of morphine CPP behavior [24]. 

In this study, we sought to expand upon this intriguing line of circRNA research in more detail at the molecular level in a rat model of heroin self-administration. Given that the frontal cortex has the highest abundance of circRNAs in both human and rodent brains [25], and is significantly involved in opioid seeking phenotypes [26,27], we profiled circRNAs from the orbitofrontal cortex (OFC) of rats following heroin self-administration. We identified heroin-associated circRNAs that are persistently regulated across multiple cohorts of biological replicate male and female rats immediately after 10 days of heroin self-administration. We conclude that heroin exposure regulates the expression level of several circRNAs in the OFC and such changes in circRNA pathways represent drug-induced neurobiological changes that could impact OFC-mediated processes and behavioral phenotypes.

## 2. Results

### 2.1. Heroin Self-Administration Regulates OFC circRNA Expression

**Figure 1:**
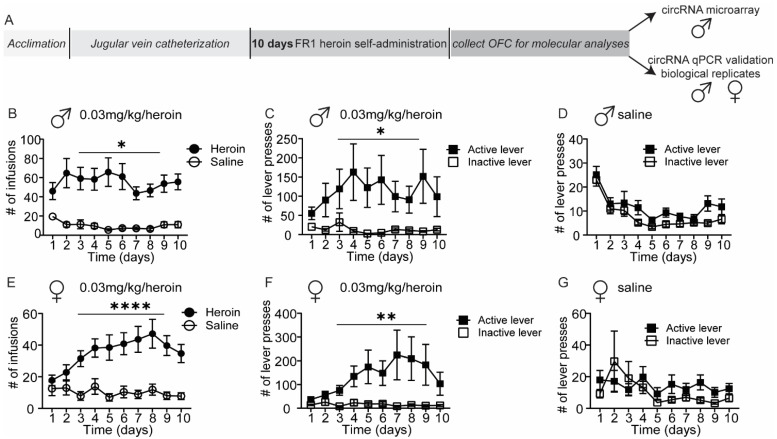
Heroin self-administration as a tool to identify heroin-responsive circRNAs in the OFC. (**A**) Schematic overview of experimental timeline. Male and female rats underwent self-administration of 0.03mg/kg/infusion of heroin or saline and were then euthanized for molecular analyses of circRNA expression in the OFC. OFC RNA from a subset of male rats was used for circRNA microarray analyses. Differentially regulated circRNAs were validated in all male rats and further examined in female rats. (**B**–**G**) Heroin and saline self-administration in male (**B**–**D**) and female (**E**–**G**) rats. Displayed are the number of infusions (**B**,**E**) and active or inactive lever presses for heroin (**C**,**F**) or saline animals (**D**,**G**). Error ± S.E.M. * *p* < 0.05; ** *p* < 0.01; **** *p* < 0.0001. Male *N* = 21/group; Female *N* = 14 saline, 13 heroin.

To identify brain circRNAs regulated by heroin exposure, we performed an unbiased circRNA expression analysis on OFC tissue from rats that underwent heroin self-administration (Figure 1). Male and female adult rats self-administered heroin (0.03 mg/kg/infusion) or saline in daily 6 h sessions for 10 days (Figure 1A). As expected, male subjects in the heroin group quickly learned the self-administration procedure and consistently received more infusions than saline animals (two-way repeated measures (RM) ANOVA, main effect of heroin availability: F (1, 38) = 26.50, *p* < 0.0001; Figure 1B). Within each experimental group, heroin and saline respectively, we compared the number of active and inactive lever presses during the entire duration of the self-administration paradigm. In the male heroin group (Figure 1C), two-way RM ANOVA revealed a main effect of active lever (F (1, 40) = 4.590, *p* = 0.0383) but no main effect was found for the factor time (F (1.210, 48.41) = 1.405, *p* = 0.2476); however, significant interaction was found between these two factors (F (9, 360) = 2.038, *p* = 0.0345). In the male saline group, no difference between the active and inactive lever was found, as expected (Figure 1D). Two-way RM ANOVA revealed no main effect of active lever (F (1, 40) = 3.432, *p* = 0.0713) but a significant main effect of time was found (F (2.629, 102.8) = 17.21, *p* = <0.0001) insofar as the subjects lost interest over time towards saline infusion and associated cues. Similarly, female rats self-administered more heroin than saline infusions (Two-way RM ANOVA, main effect of heroin availability: (F (1, 25) = 17.93, *p* = 0.0003, Figure 1E). In the female heroin group, two-way RM ANOVA revealed a main effect of active lever (F (1, 24) = 5.740, *p* = 0.0247), but not time (F (1.232, 29.57) = 2.408, *p* = 0.1262), and a significant heroin × time interaction was detected (F (9, 216) = 2.660, *p* = 0.0060 (Figure 1F). On the contrary, in the female saline group, two-way repeated measure ANOVA determined there were no main effects of active lever (F (1, 26) = 0.5578, *p* = 0.4619) or time (F (1.712, 44.52) = 2.030, *p* = 0.1494, Figure 1G). 

**Figure 2:**
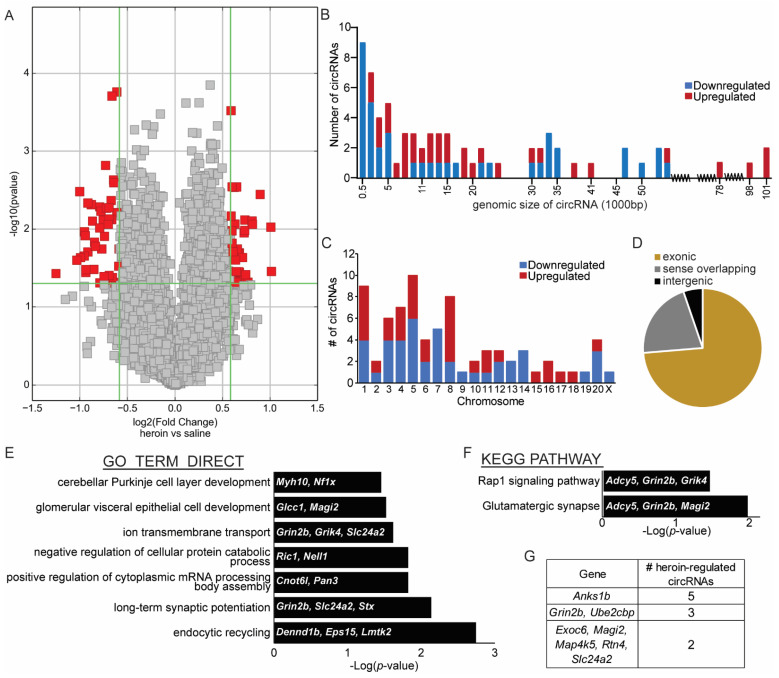
Heroin-associated circRNAs are derived from genes distributed across the genome and are mostly exonic. (**A**) Volcano plot depicting differentially expressed circRNAs in the OFC after heroin self-administration, as measured by microarray analyses. Red dots indicate circRNAs that meet statistical criteria for significantly different compared to saline. Grey dots represent circRNAs that are not statistically different between heroin and saline. (**B**) Genomic size in base pairs (bp) of each circRNA differentially expressed between heroin and saline animals, as indicated by the beginning and end position of the circRNA’s backsplice junction. (**C**). Chromosomal location of each differentially regulated circRNA. (**D**) Pie graph depicting the proportions of heroin-associated circRNAs that are exonic, intergenic, or sense overlapping. (**E**,**F**) Results from gene ontology analyses (**E**) and KEGG pathway analyses (**F**) indicating the terms significantly enriched from the gene list of linear mRNAs that give rise to differentially expressed heroin-associated circRNAs. For each term, the genes identified in the microarray analysis that belong to the term list are indicated. (**G**) List of repeat heroin-associated circRNAs that are derived from the same linear gene.

**Figure 3:**
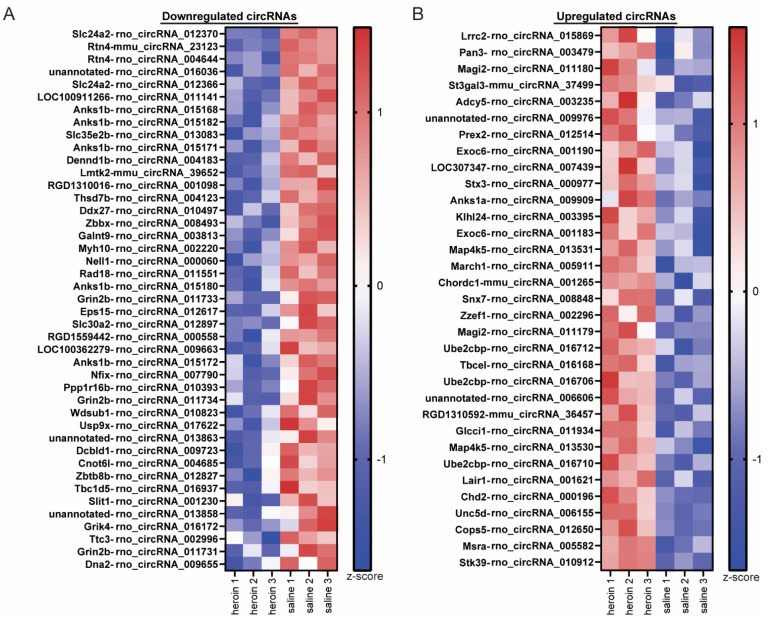
Heroin regulates OFC circRNA expression in male rats. Heatmap of downregulated (**A**) and upregulated (**B**) circRNAs in the OFC of male rats after 10 days of heroin self-administration. Z-scores of normalized intensities were used to create the heatmap. Displayed are the official circRNA names as well as the linear gene from which each circRNA is derived.

At the end of the 10th self-administration session, animals were euthanized and the OFC was collected from each animal for molecular analyses of circRNA expression as described in Figure 1A. We chose the OFC because the frontal cortex has a high circRNA abundance [25] and the OFC is critically involved in opioid-seeking phenotypes [27,28,29,30]. We first profiled circRNAs in the OFC of three male heroin and three male saline animals using a microarray analyses service provided by Arraystar, Inc. The microarray contained 14,145 probes directed against backsplice junctions of circRNAs that were previously identified in the rat nervous system [6]. The normalized intensity values obtained with the microarray were similar for the six samples used (Supplementary Figure S2). A correlation analysis between heroin and saline animal values indicated that the majority of the circRNAs examined had similar corresponding intensity between the two groups and only those that deviated from this pattern were identified as statistically significant (Supplementary Figure S3). Heroin self-administration regulated the expression of 76 circRNAs in the OFC, with 43 downregulated and 33 upregulated compared to saline control animals (Figure 2 and Figure 3 and Supplementary Table S1). Because the microarray does not reveal discrete information on the entire sequence of a given circRNA, we initially examined the genomic coordinates of the backsplice junction start and end positions for each heroin-associated circRNA. All small heroin-associated circRNAs less than 500 bp were downregulated, but in general, both up- and downregulated heroin-associated circRNAs varied in genomic length as well as chromosomal location (Figure 2C). Despite the large range in genomic size, the majority of heroin-associated circRNAs (74%) hybridized onto probes designed to target backsplice junctions from exonic circRNAs, suggesting that the actual spliced circRNA size of heroin-associated circRNAs is likely much smaller than the size indicated by the genomic coordinates of the backsplice junction (Figure 2D). A total of 21% of heroin-associated circRNAs are predicted to be intergenic, containing intronic regions.

CircRNAs are believed to function in a multitude of processes, with some capable of sponging miRNAs, binding RNA-binding proteins to regulate transcription or interact with proteins to modulate enzymatic processes. While these functions may differ greatly from the canonical functions associated with the parental gene locus that gives rise to a circRNA, it has been shown that one of the most common functions of circRNAs is to regulate parental gene splicing, therefore affecting its canonical function [31]. For this reason, we sought to identify enriched pathways associated with the genes that give rise to heroin-associated circRNAs in the OFC. Using the publicly available webtool DAVID, we were able to map 54 of the 76 heroin-associated circRNAs to known rat mRNAs. Gene ontology analyses revealed the top five enriched pathways of linear genes that give rise to heroin-associated circRNAs were: ‘endocytic recycling’; ‘long-term potentiation’; ‘positive regulation of cytoplasmic mRNA processing body assembly’; ‘negative regulation of cellular protein catabolic process’; and ‘ion transmembrane transport’ (Figure 2E). KEGG pathway analyses identified enrichment of linear genes involved in terms called ‘’glutamatergic synapse’ and ‘Rap1 signaling pathway’ (Figure 2F). The glutamate ionotropic receptor NMDA type subunit 2B (*Grin2b*) gene appeared in four of the enriched terms. As we further explored the identity of genes that are spliced following heroin self-administration, we observed significant dysregulation of multiple circRNAs derived from the same gene. Five circRNAs from ankyrin repeat and sterile alpha motif domain containing 1B (*Anks1b*) were regulated by heroin; three from *Grin2b* and ubiquitin protein ligase E3D (*Ube2cbp/Ube3d*); and two each from exocyst complex component 6 (*Exoc6*), membrane associated guanylate kinase, WW and PDZ domain containing 2 (*Magi2*), mitogen-activated protein kinase kinase kinase kinase 5 (*Map4k5*), reticulon 4 (*Rtn4*) and solute carrier family 24 member 2 (*Slc24a2*), indicating that these genes may undergo additional splicing events (Figure 2G). The full list of regulated heroin-associated circRNAs and their linear mRNAs are listed in the heatmaps in Figure 3A (downregulated) and 3B (upregulated).

### 2.2. Biological Validation of Heroin-Associated circRNAs in Male and Female Rats

**Figure 4:**
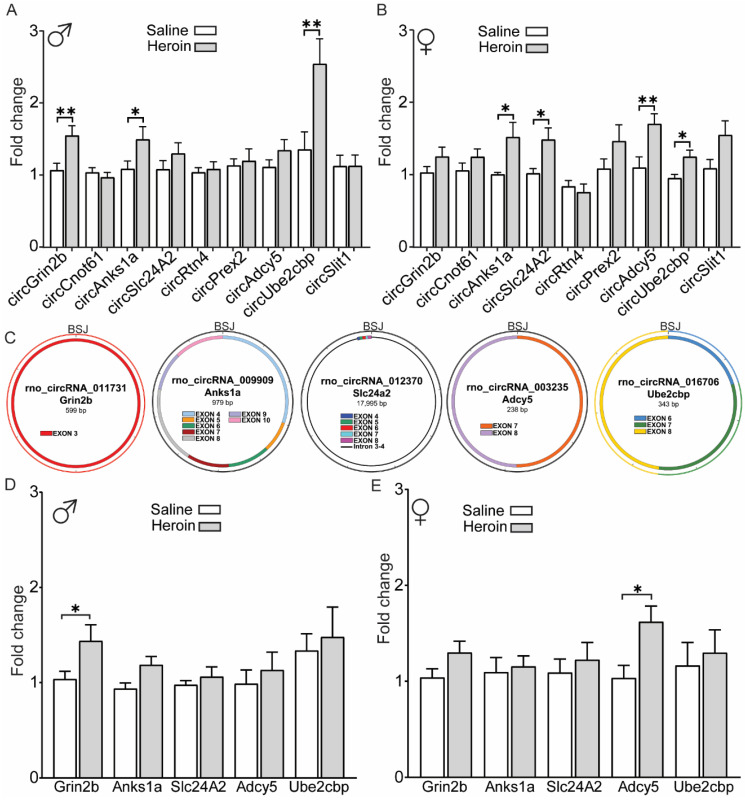
Regulation of heroin-associated circRNAs and their linear counterparts in the OFC. (**A**,**B**) qPCR validation of putative heroin-associated circRNAs in male (**A**) and female (**B**) animals after heroin or saline self-administration. (**C**) Maps of the predicted exons and introns that constitute each validated heroin-associated circRNA. BSJ—backsplice junction. (**D**,**E**) qPCR measurement of the linear mRNAs that give rise to each heroin-associated circRNA in male (**D**) and female (**E**) animals after heroin or saline self-administration. Error +/− SEM. * *p* < 0.05; ** *p* < 0.01.

To examine the accuracy of the microarray findings and the reproducibility of heroin-induced circRNA regulation, we measured expression of 10 heroin-associated circRNAs in biological replicate male and female animals after heroin self-administration using qPCR. We designed TaqMan probes that span circRNA back-splice junctions (BSJs) of 10 circRNAs: rno_circRNA_011731 (*Grin2b*), rno_circRNA_004685 (*Cnot6l*), rno_circRNA_009909 (ankyrin repeat and sterile alpha motif domain containing 1B; *Anks1a*), rno_circRNA_012370 (*Slc24a2*), rno_circRNA_004644 (*Rtn4*), rno_circRNA_012514 (phosphatidylinositol-3,4,5-trisphosphate dependent rac exchange factor 2; *Prex2*), rno_circRNA_003235 (adenylate cyclase 5; *Adcy5*), rno_circRNA_016706 (*Ube2cbp*), rno_circRNA_001230 (slit guidance ligand 1; *Slit1*), and mmu_circRNA_39652 (lemur tyrosine kinase 2; *Lmtk2*). We selected circRNAs that were derived from genes highlighted in the gene ontology and pathway analyses (Figure 2E,F) or were small in size and thus may be amenable to functional manipulations in future studies. In male animals, we observed significant upregulation of circGrin2b, circAnks1a and circUbe2cbp with qPCR (unpaired *t*-test, heroin vs. saline male animals: circGrin2b, t (37) = 3.307, *p* = 0.002; circAnks1a, t (38) = 2.070, *p* = 0.045; circUbe2cbp, t (37) = 2.718, *p* = 0.010) (Figure 4A). No statistical significance was observed for circCnot6l, circSlc24a2, circRtn4, circPrex2, circAdcy5 or circSlit1. We were unable to detect amplification for circLmtk2. In female animals that underwent self-administration, we detected significant upregulation of circAnks1a, circSlc24a2, circAdcy5 and circUbe2cbp (unpaired *t*-test, heroin vs. saline female animals: circAnks1a, t (18) = 2.433, *p* = 0.026; circSlc24a2, t (20) = 2.175, *p* = 0.042; circAdcy5, t (23) = 3.258, *p* = 0.035; circUbe2cbp, t (23) = 2.453, *p* = 0.022; Figure 4B). No significant differences were observed for circGrin2b, circCnot6l, circRtn4, circPrex2 or circSlit1 for female animals, although circSlit1 had a strong trend for upregulation (*p* = 0.053). Overall, the regulation of five putative heroin-associated circRNAs (Figure 4C) identified in the microarray analyses was replicated with two circRNAs (circAnks1a and circUbe2cbp) consistently regulated in both male and female animals after heroin self-administration. However, all of the five heroin-associated circRNAs were regulated in similar patterns in both male and female animals and two-way ANOVA analysis did not reveal any sex by treatment interactions. Indeed, statistical analysis of male and female data combined demonstrated significant regulation of all five heroin-associated circRNAs (unpaired *t*-tests, saline vs. heroin, circGrin2b: t (58) = 3.476, *p* = 0.001; circAnks1a: t (58) = 2.981, *p* = 0.004; circSlc24a2: t (44) = 2.320, *p* = 0.025; circAdcy5: t (63) = 2.654, *p* = 0.010; circUbe2cbp: t (62) = 2.975, *p* = 0.004). We further validated the existence of each circRNA by performing an RNase R digestion assay followed by qPCR for circRNA and linear mRNA pairs. circRNAs are resistant to RNase R digestion while typically many mRNAs, but not all, are rapidly degraded by RNase R treatment [15]. As expected, expression of each circRNA remained intact following RNase R digestion, while the majority of the linear mRNAs showed degradation (Supplementary Figure S4). Therefore, we conclude that the set of heroin-associated circRNAs we have validated are rat circRNAs. The predicted size and exon content of each of the five validated circRNAs is shown in Figure 4B.

### 2.3. Regulation of Heroin-Associated circRNAs May Be Independent of Host Gene Regulation

Individual circRNAs may be co-regulated with their linear counterparts or independently regulated [21], suggesting the existence of independent cellular functions. Therefore, upregulation of heroin-associated circRNAs may simply be due to more linear mRNA production. To address this, we next measured the expression of the linear mRNA counterparts of validated circRNAs to gain more insights into the specific regulation of heroin-associated circRNAs (Figure 4D,E, male and female respectively). In male animals, linear *Grin2b* was significantly elevated after heroin in the same direction of its circular transcript, rno_circRNA_011731 (unpaired *t*-test: t (25) = 2.407, *p* = 0.024, Figure 4D). However, expression of linear *Anks1a*, *Slc24a2*, *Adcy5* and *Ube2cbp* was not significantly different between male heroin and saline animals. In female animals, linear *Grin2b*, *Anks1a*, *Slc24a2* and *Ube2cbp* were not regulated by heroin, but linear *Adcy5* was significantly elevated (unpaired *t*-test: t (24) = 2.515, *p* = 0.019; Figure 4E). These results indicate that circGrin2b and circAdcy5 may be coregulated with their linear mRNA but circAnks1a, circSlc24a2 and circUbe2cbp are regulated independent of their linear mRNA.

### 2.4. circUbe2cbp Is Dually Regulated by Both Heroin and Sucrose Reward Seeking in the OFC

**Figure 5:**
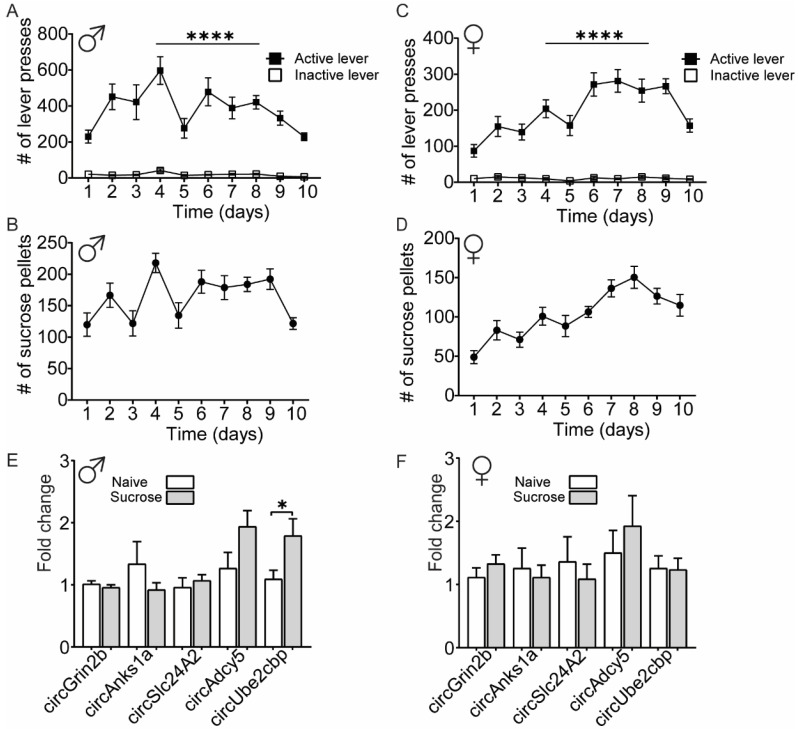
The heroin-associated circRNA circUbe2cbp is similarly regulated by sucrose self-administration in the male OFC. (**A**–**D**) Sucrose self-administration data for male (**A**,**B**) and female (**C**,**D**) animals. Shown are average lever presses (**A**,**C**) and number of sucrose rewards received (**B**,**D**) N = 18 males, 17 females. (**E**,**F**) qPCR measurement of validated heroin-associated circRNAs in male (**E**) and female (**F**) animals that self-administered sucrose or were exposed to the self-administration chamber (naïve). Error ± SEM. * *p* < 0.05; **** *p* < 0.0001.

To investigate whether the OFC circRNA expression changes detected in the heroin-exposed rats were specific for a drug reward or may generalize to an unspecific physiological response to a rewarding stimulus, we evaluated the expression level of heroin-associated circRNAs in a separate cohort of rats that underwent sucrose self-administration (Figure 5). Male and female adult rats were trained to self-administer sucrose pellets in a paradigm identical to the heroin-self-administration. Both male and female animals quickly learned to discriminate between active and inactive levers and a significant main effect of sucrose availability was observed for both sexes (two-way RM ANOVA, male sucrose: F (1, 34) = 93.02, *p* < 0.0001; female sucrose: F (1, 32) = 128.60, *p* < 0.0001; Figure 5A–D). We performed molecular analysis of circRNA expression in the OFC after the last sucrose session with qPCR. In male animals, only one circRNA, rno_circRNA_016706 (circUbe2cbp), was significantly upregulated in the OFC after sucrose self-administration (unpaired *t*-test: t (26) = 2.209, *p* = 0.036; Figure 5E). However, this change was not observed in female animals (Figure 5F) and none of the remaining heroin-associated circRNAs were significantly regulated by sucrose in the OFC of male or female animals. Taken together, these findings suggest that four of the validated heroin-associated circRNAs—circGrin2b, circSlc24a2, circAnks1 and circAdcy5—are specific for heroin reward, and one, circUbe2cbp, is regulated by both heroin and sucrose rewards in male animals only.

### 2.5. Elucidation of a circRNA-miRNA Network in the OFC after Heroin Self-Administration

**Figure 6:**
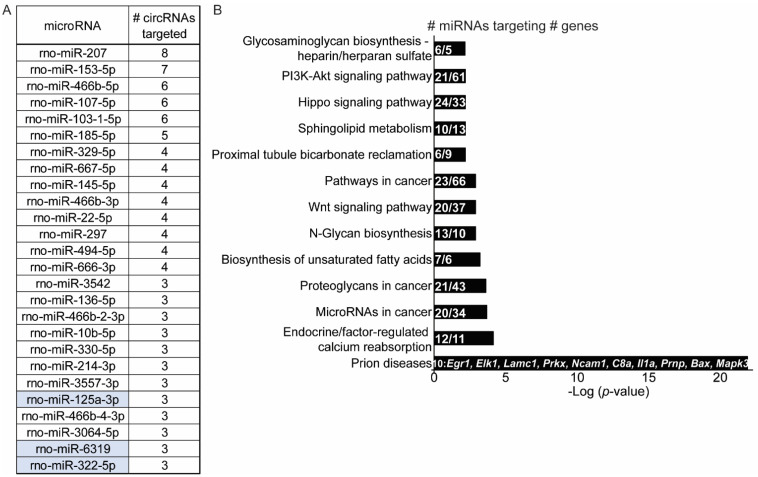
A predicted circRNA–miRNA network in the OFC associated with heroin exposure. (**A**) List of miRNAs predicted to target at least 3 heroin-associated circRNAs identified in the microarray analysis. Highlighted miRNAs target circRNAs validated with qPCR. (**B**) Pathway analysis of the target genes of miRNAs listed in (**A**). For each significant pathway, the number of miRNAs that target the pathway is listed, as well as the number of genes in the pathway that the miRNAs target.

Many circRNAs contain miRNA recognition elements (MREs) that may function to limit the role of a miRNA within a cell [32]. miRNAs are small ~18–24 nucleotide noncoding RNAs that bind target mRNA sequences with a 6-8 nucleotide ‘seed’ region contained within the miRNA [33]. Once bound, miRNAs prevent protein translation of their target mRNA by deadenylation of the mRNA poly A tail [34]. circRNA sponging of a miRNA can have far reaching consequences, as each miRNA is predicted to target 100–1000′s of mRNAs and miRNA can regulate ~60% of the proteome [35]. We examined the MREs on heroin-associated circRNAs with an analysis provided by Arraystar during the circRNA microarray. Arraystar’s proprietary tool uses an algorithm based on the Targetscan [36] and miRanda [37] to identify putative circRNA-miRNA interactions. Using this analysis, we determined the top five miRNAs predicted to target MREs on the validated heroin-associated circRNAs (Table 1). The highest number of sponge sites were observed for miRNAs targeting circAdcy5, which has three sites each predicted to bind rno-mir-15b-5p, rno-mir-16-5p, rno-mir-322-5p, rno-mir-324-3p and rno-mir-497-5p. Finally, we identified all miRNAs predicted to target at least three heroin-associated circRNAs (Figure 6A). rno-mir-207 had the most enriched MRE binding sites in our list of heroin-associated circRNAs with a total of 8. rno-mir-125a-3p, rno-mir-6319 and rno-mir-322-5p were each predicted to bind three circRNAs, including circRNAs that were validated in this study (Table 1 and Figure 6A). Pathway analysis of the predicted targets of miRNAs that have enriched MRE sites in our heroin-associated circRNA list was performed using DIANA miR-Path [38] and identified 10 pathways targeted by the miRNA list (Figure 6B). Although pathway analysis of miRNA targets remains heavily biased towards non-neuronal pathways, the most significant pathway was ‘Prion Disease’ and included gene targets previously described for their involvement in addiction such as *Bax* (BCL2 associated X, apoptosis regulator), *Egr1* (early growth response 1), *Elk1* (ETS transcription factor ELK1), *Mapk3* (*Erk1*) (mitogen-activated protein kinase 3) and *Ncam1* (Neural Cell Adhesion Molecule 1) [4,39,40,41,42,43,44,45,46,47,48].

## 3. Discussion

circRNAs represent a novel species of RNA to study in models of addiction. Although circRNAs were discovered decades ago, limited exploration into the relationship between circRNAs and drug abuse has been completed thus far. While other studies in the neuroscience field have demonstrated that circRNAs can modulate neuronal function [7,22], the extent to which circRNAs contribute to the pathophysiology of substance use disorders is largely unknown. The work presented in the current study is the first to describe heroin-induced regulation of circRNAs in the brain. 

In this study, we have identified a set of heroin-associated circRNAs consistently regulated by heroin self-administration in the OFC of adult rats. circGrin2b, circSlc24a2, circAdcy5, circAnks1 and circUbe2cbp were all identified with an unbiased microarray analysis of the circRNA profile altered by chronic heroin self-administration in male rats. We subsequently validated these results using qPCR in male and female rats to ensure reproducibility of the data. We did not detect any sex by treatment interactions when examining the expression of these putative heroin-associated circRNAs and do not conclude that regulation of the five validated circRNAs are sex-specific. Further analysis is required to examine sex-specific regulation of circRNAs after heroin exposure and accurate detection of some circRNA changes may require large sample sizes due to small effect sizes. 

At the level of linear mRNA expression, we only detected significant upregulation of *Grin2b* in male animals and upregulation of *Adcy5* in female animals. The upregulation of circGrin2b and circAdcy5 in these respective groups of animals may therefore be due to more copies of the linear isoforms of *Grin2b* and *Adcy5* being produced in response to heroin. In contrast, upregulation of circAnks1a, circSlc24a2 and circUbe2cbp was not accompanied by a change in linear mRNA levels and may be due to heroin-induced modulation of a splicing factor. Drug-induced regulation of splicing factors has been previously reported [49,50].

Overall, our analysis suggested that the changes in circRNA expression detected in the heroin-exposed rats tends to be highly specific. Out of the five heroin-associated circRNAs that were validated, only one was dually regulated by sucrose self-administration, circUbe2cbp. The overlapping regulation of circUbe2cbp suggests that this circRNA may function as a more general response to any rewarding stimuli, including palatable food rewards. In contrast, the other four circRNAs—circGrin2b, circSlc24a2, circAdcy5 and circAnks1a—were not regulated by sucrose and appear to be specific for heroin reward. Whether any of these five circRNAs are also regulated in the OFC by other drug rewards, such as alcohol or cocaine, has not yet been determined as no such study has been performed, but circUbe2cbp seems the most likely candidate for regulation by multiple rewarding stimuli. We demonstrated that heroin self-administration of a 0.03 mg/kg dosage can induce regulation of 76 circRNAs in the OFC. This dosage of heroin has been used by many labs to model drug-seeking behavior and we have previously demonstrated that animals that have higher amounts of drug intake at this dosage typically have more drug-seeking behavior after extended forced abstinence [4,51]. However, this dosage does not result in incubation of heroin craving behavior, and we expect that a higher dosage protocol (e.g., 0.075 or 1.0 mg/kg/infusion) may result in an additional unique set of circRNAs specifically associated with long-lasting drug seeking behavior. Ideally, future studies will examine the contribution of circRNAs to a variety of drug paradigms, including withdrawal, incubation, reinstatement and extinction, using a variety of drugs (e.g., psychostimulants, nicotine, alcohol) to fully delineate the contribution of unique circRNA profiles to each drug behavior. 

Interrogation of the putative microRNA pathways that may be affected by heroin-associated circRNAs revealed a list of microRNAs largely unstudied in the field of addiction neuroscience. We identified 26 microRNAs that are predicted to bind at least three heroin-associated circRNAs and an additional 12 microRNAs predicted to bind circGrin2b, circAdcy5, circAnks1a, circSlc24a2 or circUbe2cbp. Of these, let-7b-5p, which is predicted to bind circAdcy5, has been reported to be elevated in both the serum and the plasma of patients with heroin-use disorder [52,53]. Moreover, the putative targets of the heroin-associated circRNA-miRNA network include genes that have been studied extensively in the field of addiction, including *Bax*, *Egr1*, *Elk1*, *Mapk3* (*Erk1*) and *Ncam1*. Specifically in the context of opioids, abstinence from heroin self-administration upregulates *Egr1* in the frontal cortex of rats [40]. Levels of phosphor-Mapk3 (Erk1) were significantly downregulated in the prefrontal cortex of postmortem tissue from patients with opioid use disorder, although expression of *Mapk3* (*Erk1*) has also been reported to be upregulated in the locus coeruleus and striatum of rats treated with morphine [45,46]. Regulation of *Elk1* has been reported in the nucleus accumbens of rats that self-administered the same heroin dosage used in this study as well as in postmortem tissue from patients with heroin use disorder [4]. Thus, the function of circRNA–miRNA networks identified in this study may be to regulate mRNA pathways of genes that contribute to heroin-induced pathology. Further study into the complete circRNA–miRNA–mRNA pathways will illuminate a full picture of the complex interactions of these molecules in response to heroin exposure.

Our study of circRNAs regulated by heroin in the OFC is the first unbiased analysis of heroin-induced circRNA expression published to date. Microarrays represent a suitable method of circRNA measurement but are not without limitations. The probes that are used in the microarray are only designed to detect the backsplice junction of a circRNA. Because of this, no information is available regarding the true size of the circRNA detected. Many circRNAs can arise from one linear RNA and various combinations of exons or introns may be included. We have listed the genomic size of the circRNAs detected in the microarray performed here as well as the predicted size of the validated heroin-associated circRNAs including only exons that exist between the genomic coordinates. An exception to this is circSlc24a2, which contains a backsplice junction that includes an intron. Previous studies have indicated that the majority of circRNAs only contain exons and our data describing the location of the backsplice junction in Figure 2 support this notion [15]. Limited options currently exist for profiling circRNAs, but future studies utilizing sequencing technology may have the ability to detect more circRNAs and include information about the actual size or exons/introns included in the sequenced circRNAs. Many sequencing companies require at least 5 ug of RNA, as opposed to the 2.5 μg required for a microarray, which is a limitation if a researcher desires to profile individual samples from animals without pooling samples. Until the input requirement for circRNA sequencing is reduced, it is unlikely that researchers utilizing mouse tissue would be able to generate enough high-quality RNA from brain areas critically involved in drug seeking such as the nucleus accumbens, central amygdala, basolateral amygdala, ventral tegmental area, prefrontal cortex or OFC. We were able to validate half of the candidate heroin-associated circRNAs identified in the microarray with subsequent biological replicate samples of OFC tissue. Typically, RNA sequencing data has been demonstrated to be more accurate than microarrays for mRNA and microRNA measurements; thus, moving forward with the adaptation of enhanced methodologies to perform circRNA sequencing would allow more researchers to perform the most accurate methods to profile circRNAs in a variety of experimental conditions. 

The existing literature on drug-induced circRNA expression has reported circRNA regulation in primary cortical neurons and the cerebellum in response to methamphetamine treatment, and in the striatum in response to cocaine self-administration [54,55,56]. None of the five heroin-associated circRNAs validated in the present study were regulated by psychostimulants in the aforementioned studies. This may be due to differences in treatment regimens and brain region. A study on postmortem nucleus accumbens tissue from patients with alcohol use disorder identified networks of predicted circRNA–microRNA–mRNA interactions that are regulated with chronic alcohol use [57]. Intrathecal injection of the opioid morphine regulated expression of circRNAs in the spinal cord [58] and systemic treatment with morphine regulated expression of circRNAs arising from the mu-opioid receptor in the whole brain of mice [23]. A morphine conditioned place preference (CPP) paradigm regulated the expression of a circRNA pathway arising from the gene Tmeff-1 in the nucleus accumbens [24]. circTmeff-1 and its predicted sponge targets, mir-541-5p and mir-6934-3p, contribute to maintenance of morphine CPP behavior [24], suggesting that circRNA pathways may be critical regulators of drug seeking. It is likely that each drug profile will induce a unique pattern of circRNA expression changes that vary from brain region to brain region, similar to the effects that have been observed with microRNA or mRNA profiling in drug-exposed animals and human patients. While these studies have paved the way for further exploration in the role of circRNAs in addiction, systematic dissection of the contribution of individual circRNAs to the pathophysiology of drug exposure or drug seeking behaviors is a monumental task that will hopefully begin in the decades to follow.

## 4. Materials and Methods

### 4.1. Subjects

Seventy-seven adult male and fifty-six adult female Sprague Dawley rats (Charles River Laboratories), 8 weeks old, were used in this study. Animals were pair-housed on a reverse light/dark cycle (lights on at 9:00 a.m.; off at 9:00 p.m.) with constant room temperature (22 ± 2 °C) and humidity (40%). Animals were provided food ad libitum except where described. All procedures followed the National Institutes of Health’s Guide for the Care and Use of Laboratory Animals and were approved by Temple University’s Institutional Animal Care and Use Committee.

### 4.2. Drug

Diamorphine hydrochloride (heroin) was obtained from the National Institute on Drug Abuse drug supply program and dissolved in 0.9% sterile sodium chloride for drug self-administration experiments.

### 4.3. Surgery

After 5–7 days of acclimation, animals underwent intravenous catheter surgery for heroin or saline self-administration, as previously described [51]. Following surgery, all animals were singly housed for the remainder of the study. Twenty-four hours prior to the initiation of self-administration, catheter patency was tested with propofol (1%) and animals were food restricted. Catheters were flushed daily with heparinized saline before and after each self-administration session. If there was resistance, the catheter patency was re-tested on an individual and as needed basis. To ensure the correct amount of heroin was self-administered, the volume in each syringe was noted before and after each session and checked against the number of lever presses. Food-restriction during self-administration did not decrease bodyweight and was used to increase motivation to perform the task (Supplementary Figure S1).

### 4.4. Self-Administration

Heroin self-administration studies were conducted in operant chambers under a fixed ratio (FR) 1 schedule, as previously described [51]. For heroin self-administration, active lever pressing resulted in activation of the infusion pump for intravenous infusion of 0.03 mg/kg/infusion of heroin solution, presentation of a 65 db, 2.9 kHz acoustic cue and illumination of a stimulus light above the active lever. A 20-s time out period followed each drug infusion. Animals were trained for 10 days in 6 h daily sessions. A separate group of rats underwent saline self-administration and did not receive heroin infusions. For sucrose self-administration, uncatheterized animals underwent a similar protocol during 2 h daily sessions for 10 days, except active lever pressing resulted in delivery of a 45 mg chocolate-flavored sucrose pellet (Bio-Serv, Flemington, NJ, USA). To control for experience of exposure to the self-administration chamber without catheterization, sucrose animals were compared to a group of naïve animals that were placed in the self-administration chamber but did not undergo self-administration of any reward.

### 4.5. Tissue Collection and RNA Extraction

Immediately after the 10th self-administration session, all animals were briefly anesthetized with 5% isoflurane and rapidly decapitated. Brains were removed and frozen in ice-cold isopentane on dry ice. Brains were kept at −80 °C until dissection. The orbitofrontal cortex (OFC) was dissected with a 1mm sample corer on a cold plate maintained at −20 °C to ensure tissue remained frozen. Frozen tissue punches were stored at −80 °C until RNA extraction. Total RNA was extracted from OFC tissue using the MIRvana Paris Protein & RNA Isolation System (Thermo Fisher Scientific, Waltham, MA, USA) according to manufacturer’s instructions as previously described [59]. The RNA fraction was suspended in RNase free water.

### 4.6. Microarray Analysis

Detection of differentially expressed circRNAs was achieved with microarray analyses performed by Arraystar Inc. (Rockville, MD). A total of 2.5 μg of pure RNA from 8 individual male OFC samples was submitted for analysis. Purity and concentration of total RNA samples were determined using a NanoDrop ND-1000 (Thermo Fisher Scientific, Waltham, MA, USA). RNA integrity was assessed by electrophoresis on a denaturing agarose gel. Sample labeling and array hybridization were performed according to the manufacturer’s protocol (Arraystar Inc., Rockville, MD, USA). Briefly, total RNAs were digested with RNase R (Lucigen, Middleton, WI, USA) to remove linear RNAs and enrich circular RNAs. Then, the enriched circular RNAs were amplified and transcribed into fluorescent cRNA utilizing a random priming method (Arraystar Super RNA Labeling Kit; Arraystar, Inc., Rockville, MD, USA). The labeled cRNAs were purified by RNeasy Mini Kit (Qiagen). The concentration and specific activity of the labeled cRNAs (pmol Cy3/μg cRNA) were measured by NanoDrop ND-1000. A total of 1 μg of each labeled cRNA was fragmented by adding 5 μL 10 × Blocking Agent and 1 μL of 25 × Fragmentation Buffer, then heated the mixture at 60 °C for 30 min, and finally 25 μL 2 × Hybridization buffer was added to dilute the labeled cRNA. In total, 50 μL of hybridization solution was dispensed into the gasket slide and assembled to the circRNA expression microarray slide. The slides were incubated for 17 h at 65 °C in an Agilent Hybridization Oven. The hybridized arrays were washed, fixed and scanned using the Agilent Scanner G2505C. Scanned images were imported into Agilent Feature Extraction software for raw data extraction. Quantile normalization of raw data and subsequent data processing were performed using the R software limma package. After quantile normalization of the raw data, low intensity filtering was performed, and the circRNAs that had at least 3 out of 12 samples ‘present’ were retained for further analyses. Statistical significance was estimated by *t*-test. circRNAs having fold changes >1.5 and *p*-values < 0.05 were selected as significantly differentially expressed. A proprietary algorithm owned by Arraystar was used to predict miRNA:circRNA interactions. All raw microarray data have been deposited to the Gene Expression Omnibus repository under record GSE189192 and descriptive statistics of microarray data can be found in Supplemental Table S1.

### 4.7. Quantitative Polymerase Chain Reaction (qPCR)

qPCR measurement of differentially regulated circRNAs or their linear counterparts was performed using a Quantstudio3 qPCR machine (Thermo Fisher Scientific, Waltham, MA, USA). A total of 500 ng of total RNA was reverse transcribed into cDNA using 200 units of Maxima Reverse Transcriptase (Thermo Fisher Scientific, Waltham, MA, USA), 20 units of RiboLock RNase inhibitor (Thermo Fisher Scientific, MA), 0.5 μM dNTP mix (Thermo Fisher Scientific, Waltham, MA, USA), 100 pmol random hexamer primers (Thermo Fisher Scientific, Waltham, MA, USA) and 4 μL 5X RT Maxima RT buffer (Thermo Fisher Scientific, Waltham, MA, USA). Reverse transcriptase reactions were incubated at 25 °C for 10 min, 50 °C for 30 min and inactivated at 85 °C for 5 min. cDNA was diluted 1:10 then used as a template in qPCR reactions with IDT PrimeTime Gene Expression Mastermix and IDT PrimeTime qPCR Probe Assays (Integrated DNA Technologies, IDT, Coralville, Iowa). Beta actin and glyceraldehyde 3-phosphate dehydrogenase (*Gapdh*) were use as endogenous controls. Expression levels were calculated using the 2^−ΔΔCt^ method [60]. A full list of primers can be found in Supplementary Tables S2 and S3.

### 4.8. RNase R Digestion

Poly(A) tailing followed by RNase R digestion was performed as described [61] with minor changes. A total of 10 μg of rat OFC RNA was subjected to poly(A) tailing using a Poly(A) Tailing Kit (Thermo Fisher Scientific) following manufacturer’s instructions, for 10 min at 37 °C with the addition of 1 μL of RiboLock RNase inhibitor (40 U/μL; Thermo Fisher Scientific). Poly(A) RNA was extracted with acid-chloroform, ethanol precipitated and dissolved in nuclease-free water. Then, 1 ug poly(A) RNA was incubated at 37 °C for 30 min with 10× RNase R buffer (0.2 M Tris–HCl (pH 8.0), 1 mM MgCl_2_ and 1 M LiCl), 1 μL of Ribolock RNase inhibitor, and 3U of RNase R enzyme. Following RNase R digestion, reactions were immediately reverse transcribed for qPCR as described above.

### 4.9. Pathway Analyses

Gene ontology pathway analyses were performed on the list of linear mRNA genes from which heroin-associated circRNAs are derived, using the DAVID Bioinformatics Database v6.8 developed by the Laboratory of Human Retrovirology and Immunoinformatics at NIH [62,63]. Analysis identified gene ontology terms enriched in the list of linear mRNA genes attributed to heroin-associated circRNAs, using the GOTERM_BP_DIRECT collection of terms. KEGG Pathway analysis was also performed to further identify enriched pathways in the gene list with a *p*-value of less than 0.05. Pathway analysis of predicted miRNA targets was performed using DIANA mirPath v3 software [38]. A ‘pathways union’ analyses was performed to identify common pathways targeted by miRNAs that are predicted to bind at least three heroin-associated circRNAs, with a *p* value of less than 0.001.

### 4.10. Statistical Analysis

Mean data are presented, with error bars indicating the standard error of the mean (SEM). Two-way analysis of variance (ANOVA) with repeated measures (RM) was performed on self-administration infusion data by using heroin or saline availability as the between-subject factors and time as within-subject factor. Two-way RM ANOVA was used to compare self-administration of the active versus inactive lever over 10 days. D’Agostino normality tests were performed on all datasets. Unpaired Student’s *t*-tests were used to analyze differences between two groups with normal distributions. Nonparametric Mann–Whitney tests were performed to compare differences between two groups without a normal distribution. A *p*-value of less than 0.05 (*p* < 0.05) was considered statistically significant. Statistical analyses of qPCR data were performed on ΔΔCT values prior to log transformation of fold change. GraphPad software was used for all analyses (Prism version 9; GraphPad, San Diego, CA, USA).

## Figures and Tables

**Table 1:** microRNAs predicted to bind validated heroin-associated circRNAs. Shown are the locations of the top 5 predicted MRE sponge sites for each validated heroin-associated circRNA as well as the miRNAs predicted to bind at each MRE sponge site. Seed binding indicates the number of nucleotides (e.g., −6mer = 6 nucleotides) in the miRNA seed sequence that are a match for the circRNA MRE sequence. The 7mer-m8 contains the best possible 7 nucleotide binding pattern, as it includes a seed match to the 8th nucleotide in the miRNA seed region.

circRNA	Linear Gene	miRNA	# Sponge Sites	Location (bp)	Seed Binding
rno_circRNA_003235	*Adcy5*	rno-let-7a-5p	2	17 to 38	6mer
				72 to 92	8mer
		rno-let-7b-5p	2	17 to 38	6mer
				72 to 92	8mer
		rno-let-7c-5p	2	17 to 38	6mer
				72 to 92	8mer
		rno-let-7f-5p	2	17 to 38	6mer
				72 to 92	8mer
		rno-let-7i-5p	2	17 to 38	6mer
				72 to 92	8mer
rno_circRNA_009909	*Anks1a*	rno-mir-15b-5p	3	149 to 171	7mer-m8
				245 to 270	7mer-m8
				912 to 933	7mer-m8
		rno-mir-16-5p	3	149 to 171	7mer-m8
				249 to 270	7mer-m8
				913 to 933	7mer-m8
		rno-mir-322-5p	3	149 to 171	7mer-m8
				244 to 270	7mer-m8
				912 to 933	7mer-m8
		rno-mir-324-3p	3	294 to 314	7mer-m8
				577 to 598	8mer
				872 to 893	7mer-m8
		rno-mir-497-5p	3	150 to 171	7mer-m8
				245 to 270	7mer-m8
				910 to 933	7mer-m8
rno_circRNA_011731	*Grin2b*	rno-mir-26b-3p	1	144 to 168	7mer-m8
		rno-mir-100-3p	1	33 to 53	7mer-m8
		rno-mir-350	1	117 to 140	Imperfect
		rno-mir-382-5p	1	149 to 171	8mer
		rno-mir-463-3p	1	17 to 40	7mer-m8
rno_circRNA_012370	*Slc24a2*	rno-mir-125a-3p	2	344 to 365	Offset 6mer
				451 to 473	7mer-m8
		rno-mir-127-5p	2	56 to 75	8mer
				206 to 228	7mer-m8
		rno-mir-433-3p	2	128 to 149	Offset 6mer
				535 to 553	Offset 6mer
		rno-mir-6315	1	623 to 647	7mer-m8
		rno-mir-6319	1	101 to 121	8mer
rno_circRNA_016706	*Ube2cbp*	rno-mir-17-5p	1	112 to 137	7mer-m8
		rno-mir-20a-5p	1	112 to 137	7mer-m8
		rno-mir-20b-5p	1	112 to 137	7mer-m8
		rno-mir-93-5p	1	112 to 137	7mer-m8
		rno-mir-106b-5p	1	114 to 137	7mer-m8

## Data Availability

Data that support the findings in this study are available from the authors upon reasonable request.

## Supplementary Materials

The following are available online at https://www.mdpi.com/article/10.3390/ijms23031453/s1.
