# Supplementary material for: Heroin Regulates Orbitofrontal Circular RNAs
Source: Int J Mol Sci. 2022 Jan 27;23(3):1453. doi: 10.3390/ijms23031453 (PMC8836038; doi:10.3390/ijms23031453)
Supplement: Supplementary file 1 [file ijms-23-01453-s001.zip › Supplemental material- updated.pdf]

Supplemental material for ‘Heroin regulates orbitofrontal circular RNAs’ by Floris et al.

Supplemental figures:

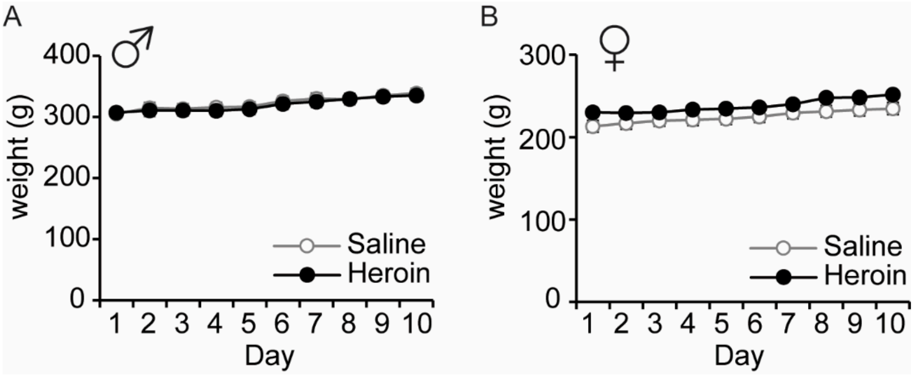

Figure S1: Body weight measurements of animals that underwent saline or heroin self-administration. Average daily body weight in grams (g) for male (A) and female (B) animals across all 10 days of self-administration.

Table S2: List of circular RNA primers used in qPCR experiments.

rno_circRNA_003235 (Adcy5)	Probe	5'-/56-FAM/ AGTTGAGTG/ZEN/TGGCCTTGGTGATGT /31ABkFQ/-3'
	Primer 1	5'-GCAACCAGGTGTCCAAG-3'
	Primer 2	5'-TGGCTCCACCTCAT AGTC-3'
rno_circRNA_009909 (Anks1a)	Probe	5'-/56-FAM/AGGGCTGTC/ZEN/TCGTTGTCGTTGTTC/31ABkFQ/-3'
	Primer 1	5'-CAGCAGGGCCTTCACT AC-3'
	Primer 2	5'-CTCACAGCAGAGACCAAGAA-3'
rno_circRNA_004685 (Cnot61)	Probe	5'-/56-FAM/CCTTTGGCA/ZEN/TCCCT ATT AGTCTGTT AAACC/31ABkFQ/-3'
	Primer 1	5'-TGT AAATTCTGCGAGGGTCTG-3'
	Primer 2	5'-GAACTTGGCCGGCTCTTC-3'
rno_circRNA_011731 (Grin2b)	Probe	5'-/56-FAM/TGGAAGAAC/ZEN/ ATGGAGGACTCATCCCT /31ABkFQ/-3'
	Primer 1	5'-CAAGAGCAGTTGCT ACAACAC-3'
	Primer 2	5'-TTCGAT AGACGGGCCAAAC-3'
rno_circRNA_012514 (Prex2)	Probe	5'-/56-FAM/ AACTTGTCC/ZEN/ ATGACATCCCTCCCA/31ABkFQ/-3'
	Primer 1	5'-CAGCATGTGCAGTGAGAGAG-3'
	Primer 2	5'-TCGATAATGGCCAAGGTGTTG-3'
mmu_circRNA_23123 (Rtn4)	Probe	5'-/ 56-FAM/CCCTGTCAG /Z EN/CTGT A TTGTCAGCAGA/31ABkFQ/-3'
	Primer 1	5'-TCAGATGCAGCAGGAAGAG-3'
	Primer 2	5'-GACACAGAGAAAGAGGACAGAT-3'
rno_circRNA_012366 (Slc24a2)	Probe	5'-/ 56-FAM/ AGA TGTTGC/Z EN/TTCCAACGGAGCTGG /31ABkFQ/-3
	Primer 1	5'-TGTCAT AGTGGCCCGAAA-3'
	Primer 2	5'-CCTTCTTCAGTCATTGTGTCAT-3'
rno_circRNA_001230 (Slit1)	Probe	5'-/56-FAM/TTGTTGTTC/ZEN/AGTCGTCAGGGTCCG/31ABkFQ/-3'
	Primer 1	5'-CAGCTGGAGTCTGTCAGAAG-3'
	Primer 2	5'-CCAGTGGCCTCT AGGA TTG-3'
rno_circRNA_016706 (Ube2cbp)	Probe	5'-/56-FAM/ ACGCCATCA/ZEN/ AAGTCCTCT ACCAGC/31ABkFQ/-3'
	Primer 1	5'-GAAGCTGACTCTGGCTCTG-3'
	Primer 2	5'-GAAACTTCCCTCAGATGGCT-3'

Table S3: List of linear mRNA primers used in qPCR experiments.

linear ActB	Probe	5'-/ 56-FAM/TCCTGGGT A/Z EN/TGGAA TCCTGTGGC/31ABkFQ/-3'	
	Primer 1	5'-TCACT ATCGGCAATGAGCG-3'	
	Primer 2	5'-GGCAT AGAGGTCTTT ACGGATG-3'	
	Assay ID:		
linear Adcy5	Probe	5'-/56-FAM/ACTGCTCGC/ZEN/CTTGACTTCCTCTG/31ABkFQ/-3'	Exons 17-18
	Primer 1	5'-ATCATCTCTGTCTTCGTGCTG-3'	
	Primer 2	5'-CTCCTCCATCTCCTCCTTCTC-3'	
	Assay ID:	Rn.PT.58.37585706	
linear Anks1a	Probe	5'-/56-FAM/CTCT ACAAC/ZEN/GCTGTGTCCTGCCAT /31ABkFQ/-3'	Exon 9-10
	Primer 1	5'-CTTGATTCTGACATCCTCGCT-3'	
	Primer 2	5'-CTTGTTCACGGAGTCTCTGTC-3'	
	Assay ID:	Rn.PT.58.46149742	
linear Gapdh	Probe	5'-/ 56-FAM/CACACCGAC/Z EN/CTTCACCA TCTTGTCT /31ABkFQ/-3	Exons 1-3
	Primer 1	5'-TCTCTGCTCCTCCCTGTTC-3'	
	Primer 2	5'-GT AACCAGGCGTCCGATAC-3'	
	Assay ID:	Rn.PT.58.35727291	
linear Grin2b	Probe	5'-/ 56-FAM/TCTGCCTTC/Z EN/TT AGAGCCA TTCAGCG /31ABkFQ/-3'	Exons 7-8
	Primer 1	5'-GCATCAGTGTCATGGTATCTCG-3'	
	Primer 2	5'-CACAAACATCATCACCCACAC-3'	
	Assay ID:	Rn.PT.58.9183827	
linear Slc24a2	Probe	5'-/56-FAM/CACACCCTT /ZEN/GACCCACTCGCT /31ABkFQ/-3'	Exons 3-5
	Primer 1	5'-GAACAGCATCTTCCAGCTCAT-3'	
	Primer 2	5'-CTCCCTTCTTCAGTCATTGTGT-3'	
	Assay ID:	Rn.PT.58.35386005	
linear Ube2cbp	Probe	5'-/56-FAM/TTAACCCTG/ZEN/CCCTCCACAACCTG/31ABkFQ/-3'	Exons 8-9
	Primer 1	5'-AACGCCATCAAAGTCCTCTAC-3'	
	Primer 2	5'-GTTCCTCGATAGTATCAACAGCA-3'	
	Assay ID:	Rn.PT.58.8524089	

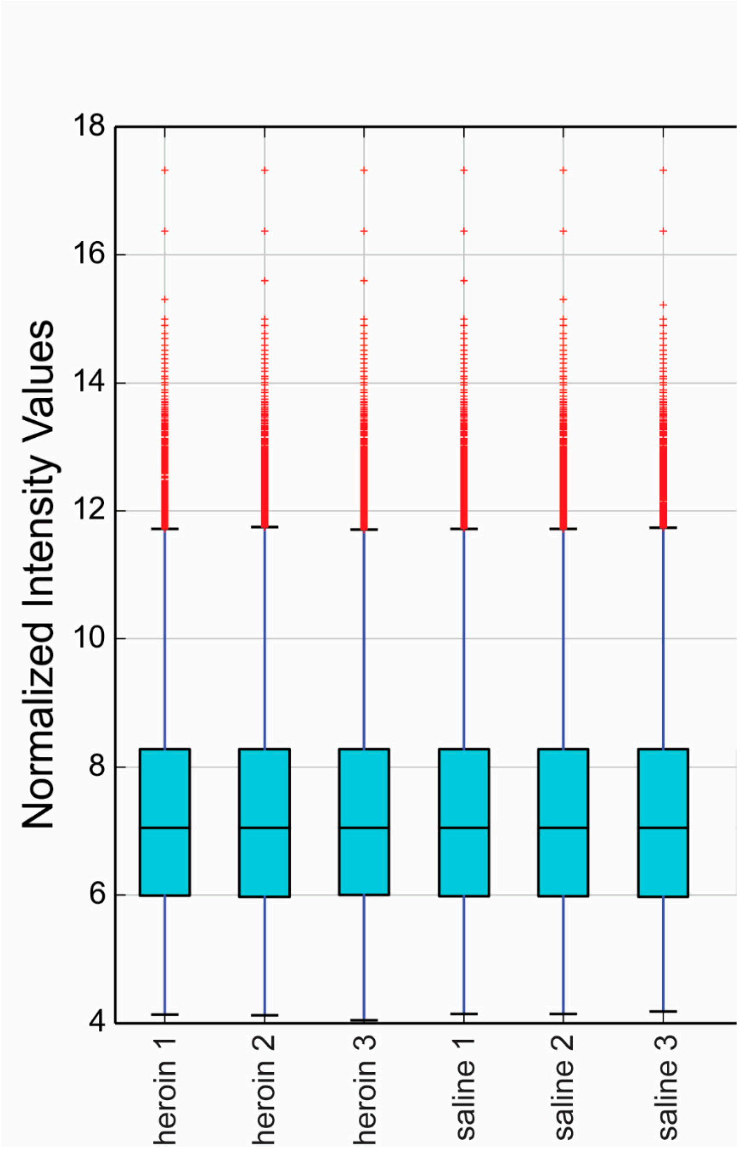

Figure S2: Box-plot of samples used for microarray analyses

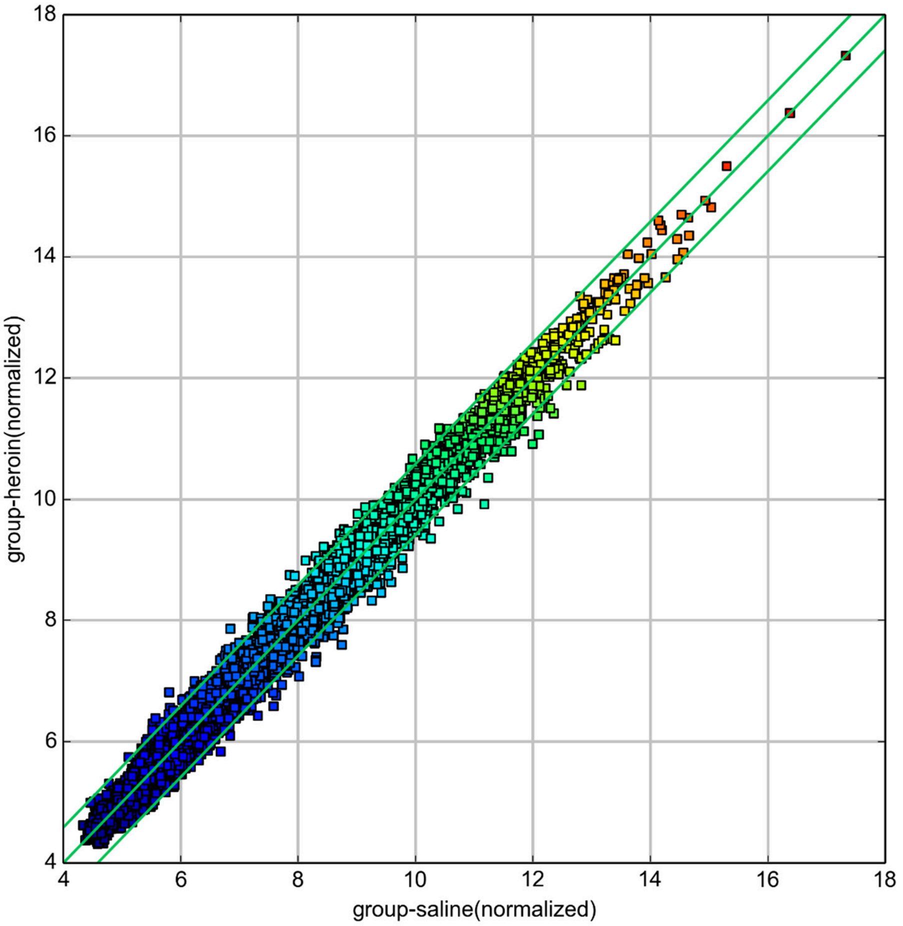

Figure S3: Correlation analyses of circRNA expression between male heroin and saline animals. Scatterplot analysis of OFC circRNA expression from the microarray between saline and heroin animals. A majority of circRNAs detected by the microarray were not regulated by treatment group (between two outer green lines), while some circRNAs (shown outside green lines) were significantly regulated between treatment groups. circRNAs expressed at lower levels are represented by darker blue squares while more highly expressed circRNAs are represented by orange and red squares.

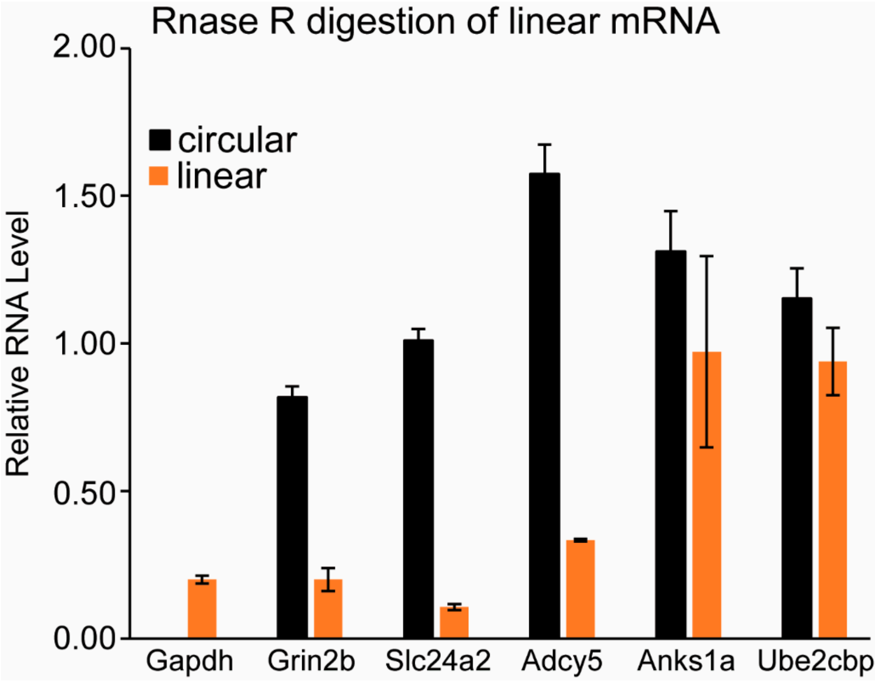

Figure S4: Validation of RNase R resistance of putative heroin-associated circRNAs: Due to their unique structure circRNAs, unlike linear mRNAs, are resistant to Rnase R degradation. Graph depicts the relative levels of both circ- and linear mRNA from samples that underwent RNase R (RNase R +) and their respective negative controls (RNase R -).
